# Enrichment and proteomic analysis of plasma membrane from rat dorsal root ganglions

**DOI:** 10.1186/1477-5956-7-41

**Published:** 2009-11-05

**Authors:** Xia Xiong, Sha Huang, Hai Zhang, Jianjun Li, Jianying Shen, Jixian Xiong, Yong Lin, Liping Jiang, Xianchun Wang, Sonping Liang

**Affiliations:** 1 College of Life Sciences, Hunan Normal University, Changsha 410081, PR China

## Abstract

**Background:**

Dorsal root ganglion (DRG) neurons are primary sensory neurons that conduct neuronal impulses related to pain, touch and temperature senses. Plasma membrane (PM) of DRG cells plays important roles in their functions. PM proteins are main performers of the functions. However, mainly due to the very low amount of DRG that leads to the difficulties in PM sample collection, few proteomic analyses on the PM have been reported and it is a subject that demands further investigation.

**Results:**

By using aqueous polymer two-phase partition in combination with high salt and high pH washing, PMs were efficiently enriched, demonstrated by western blot analysis. A total of 954 non-redundant proteins were identified from the plasma membrane-enriched preparation with CapLC-MS/MS analysis subsequent to protein separation by sodium dodecyl sulfate polyacrylamide gel electrophoresis (SDS-PAGE) or shotgun digestion. 205 (21.5%) of the identified proteins were unambiguously assigned as PM proteins, including a large number of signal proteins, receptors, ion channel and transporters.

**Conclusion:**

The aqueous polymer two-phase partition is a simple, rapid and relatively inexpensive method. It is well suitable for the purification of PMs from small amount of tissues. Therefore, it is reasonable for the DRG PM to be enriched by using aqueous two-phase partition as a preferred method. Proteomic analysis showed that DRG PM was rich in proteins involved in the fundamental biological processes including material exchange, energy transformation and information transmission, etc. These data would help to our further understanding of the fundamental DRG functions.

## Background

Dorsal root ganglion (DRG) neurons are primary sensory neurons that conduct neuronal impulses related to pain, touch and temperature senses. These primary sensory neurons send their axons peripherally to the body surface, muscles and viscera. They enter the central ascending pathways carrying information to the brain, and encode sensory messages in the form of a series of action potentials. For example, pain pathways begin with DRG that produce nociceptive signals and convey them centrally [1,2]. Shen *et al *[3] carried out morphologic analysis of normal human lumbar dorsal root ganglia by 3D magnetic resonance (MR) imaging, and the results showed that the width and length of DRG from L1 to L5 arrange from 3.23 to 6.22 mm and from 4.14 to 11.55 mm, respectively. To gain a global view of the changes in gene expression in DRGs after peripheral axotomy, Xiao *et al*. (2002) [4] carried out cDNA array on the genes mainly made from the cDNA libraries of lumbar DRGs of normal rats and of rats 14 days after peripheral axotomy. Their results showed that, of the 7523 examined genes and expressed sequence tags (ESTs), the expression of 122 genes and 51 expressed sequence tags were strongly changed, indicating the presence of molecular alterations. These genes encompass a large number of proteins from distinct families, including neuropeptides, receptors, ion channels, signal transduction molecules, synaptic vesicle proteins and others. Recently, Komori *et al *[5] have conducted a proteomics study of rat 4th and 5th lumbar (L4 and L5) DRGs after L5 spinal nerve ligation, and found 67 proteins that were tightly regulated by nerve ligation. Up to now, despite the investigation of some proteins of DRG cells, a comprehensive profile for proteome of DRG PM has not been available and is still a subject to be understood. PM of DRG cells plays important roles in cell functions. Due to the presence of specific membrane proteins, PM works as a selectively permeable barrier and communication interface of cells. Systematical characterization of the proteins in DRG PM will contribute to a better understanding of the functions of DRG cells.

For the proteomic analysis of PM, purification of PM is the crucial first step. Traditional density gradient centrifugation method is mostly used. However, due to the overlapping densities, PM preparations prepared by this method are often heavily contaminated with mitochondrial and other cellular inner membranes [6]. Additionally, this method is not well suitable for a small amount of sample [7]. Therefore, for purification of PM from DRGs that are generally difficult to isolate in large amounts, two-phase partition method may be a better choice, which was widely used in 1980's because it was believed to be able to separate mitochondria and other cellular inner membranes from PMs on the basis of their different surface properties. In an aqueous two-phase partition system, PMs tend to enter the polyethylene glycol (PEG)-rich upper phase whereas mitochondria and other subcellular organelles tend to enter the dextran-rich bottom phase [8,9]. Using high salt and high pH solutions to treat the PM debrises prepared with two-phase partition can improve PM preparation purity further, because high salt and high pH solution washing reduce non-covalent protein-protein interactions, thus lowering the amount of proteins that adsorbed to PMs [10]. In addition, electron micrographs reveal that high pH prevents the resealing of membrane structures after mechanical agitation, favoring the presence of membrane 'sheets' with free edges [11].

For the separation and identification of proteins in complex mixtures, the traditional method has been a combination of two-dimensional PAGE (2D-PAGE) and MS or MS/MS analysis of the visualized protein spots. The bias of 2D-PAGE against proteins with extreme isoelectric points and molecular weights, as well as its difficulty to resolve membrane proteins, has been well documented [12]. The hydrophobic nature of integral membrane proteins is one of the factors limiting the application of 2D-PAGE, although extensive efforts, such as the use of detergents, have been made to improve this separation [13]. One-dimensional SDS-PAGE, replacing 2D-PAGE, was used as the main protein separation method in the proteomics analysis of PM, because one-dimensional SDS-PAGE offers minimal protein loss and the resolution is sufficient to separate PM proteins [14].

The in-solution digestion in shotgun proteomics provides advantages for overcoming the limitations of gel separation of hydrophobic membrane proteins and permitting of adding a certain concentration of detergents or organic solvents to improve the denaturation, dissolution and proteolysis of membrane proteins. Therefore, the shotgun strategy was widely used in recent proteomic analysis of cell membranes [15]. In the present study, the PMs of rat DRG cells were significantly enriched using an aqueous polymer two-phase partition-based method, and the PM proteome was identified by SDS-PAGE- and shotgun-CapLC-MS/MS strategies. The obtained preliminary PM proteomic data of rat DRG would help to our understanding of the fundamental functions of DRG and improve the proteomics on DRG and other related researches.

## Results

### Enrichment of rat DRG PM

**Figure 1:**
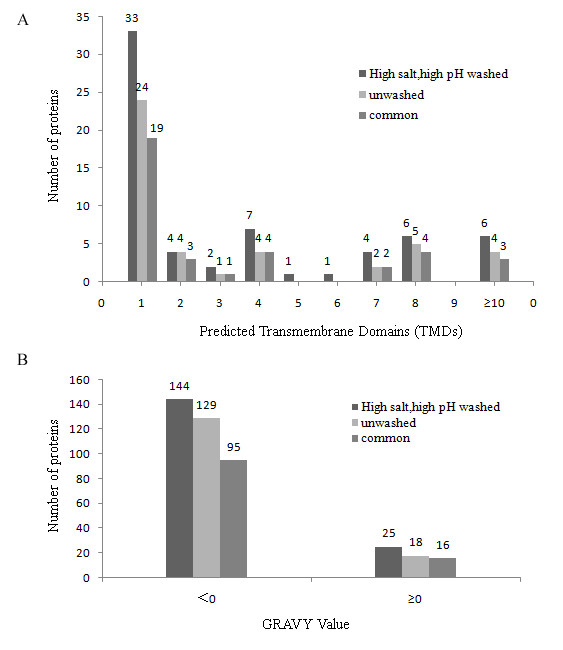
**Comparison of the PM proteins identified based washing and unwashing methods**. High salt and high pH washed, PM proteins identified from the PM fraction purified by aqueous two-phase partition followed by high salt and high pH solutions washing; Unwashed, PM proteins identified from the PM fraction purified by aqueous two-phase partition, without high salt and high pH solutions washing; Common, PM proteins identified in both of the two methods. GRAVY, grand average of hydropathicity. Negative GRAVY and positive GRAVY values indicate hydrophilic and hydrophobic proteins, respectively.

Due to the low abundance of DRG PM proteins, enrichment of PM is essential for the proteomic analysis of rat DRG PM. In this study, we first used differential velocity centrifugation to obtain crude PM fraction (CPMF). Although contaminated with some subcellular organelles, the PM was enriched to some extent. The PM in CPMF was further enriched by an aqueous two-phase system composed of Dextran T500 and PEG 3350. For evaluation of the efficiency of washing PMs with high pH and high salt solutions, we made a comparative study. The results showed that after washing the percentage of cytoplasm proteins in the total identified proteins decreased from 17.0% to 10.3%, and the content of cytoskeleton proteins was also decreased a few percentages. Particularly, the identification of integral membrane proteins was significantly improved (Fig. 1). It could be found that the number of identified PM proteins with one or more transmembrane domains (TMDs) and positive grand average value of hydropathicity (GRAVY) value were increased, demonstrating that high salt and high pH solution washing treatment was in favour of the identification of more hydrophobic integral plasma membrane proteins.

**Figure 2:**
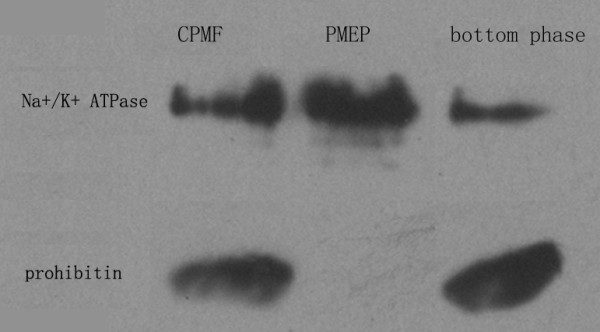
**Western blotting analysis of Na^+^/K^+ ^ATPase and Prohibitin in various rat DRG PM fractions**. PMEP, plasma membranes-enriched preparation; CPMF, crude PM fraction; BP, fraction in the bottom phase of two-phase system.

The total effectiveness of PM enrichment by 6.4% polymer two-phase system plus high pH and high salt washing was evaluated by western-blotting-based marker enzyme assay (Na^+^/K^+ ^ATPase for PM and prohibitin for mitochondria). Fig. 2 shows that, judging by the relative content of marker enzyme, CPMF contained a certain amounts of PM and mitochondria, and the concentration of PM in plasma membranes-enriched preparation (PMEP) is about 3 times that in CPMF, indicating that aqueous two-phase partitioning improved the purity of PM preparation significantly. At the same time, the marker enzyme prohibitin of mitochondria in PMEP could not be detected under the same experimental conditions, suggesting that the content of mitochondria in PMEP was very low, which was due to that mitochondria preferentially entered the bottom phase during aqueous two-phase partitioning. This was supported by the result in Fig. 2 that the content of mitochondrion in bottom phase (BP) was much higher than that of PM.

### Characterization of the identified proteins in PMEP

**Figure 3:**
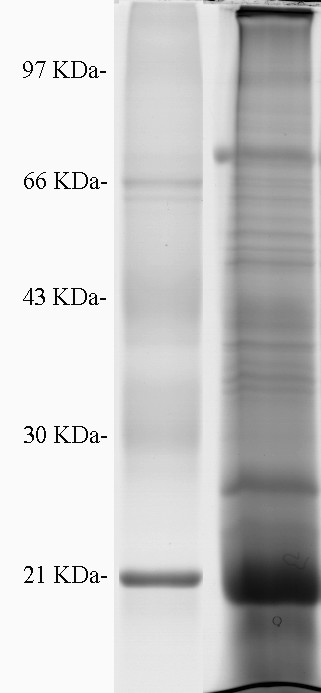
**One-dimensional SDS-PAGE image of the plasma membranes-enriched preparation (PMEP) proteins**. Molecular mass marker shown on the left.

**Figure 4:**
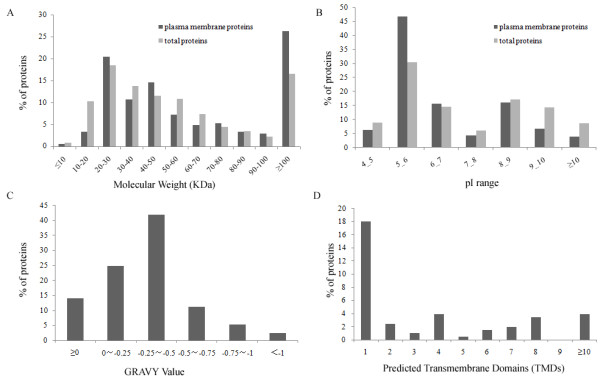
**Physicochemical characterization of proteins identified in rat DRG PM preparation**. a) Calculated molecular weight; b) Calculated isoelectric point; c) GRAVY value calculated using ProtParam algorithm; d) TMD number predicted by TMHMM.

In SDS-PAGE-based strategy, the proteins in PMEP were separated by one-dimensional SDS-PAGE (Fig. 3), and in shotgun-based strategy the proteins were directly digested in solution. After tryptic digestion and MS analysis, totals of 954 non-redundant proteins were identified from PMEP, of which 620 and 334 were identified with SDS-PAGE-based and shotgun-based strategies, respectively (see Additional file 1). We eliminated the false positive identification by using the reversed sequence databases search strategy. In order to detect the possible analytical bias in protein identification with our strategies, we analyzed the distribution of main physicochemical characteristics (molecular mass, pI and GRAVY value, etc.) of the identified proteins. Of the 954 identified proteins, 621(65.1%) were in the range of 10-60 kDa, which is the molecular mass distribution typically seen with 2DE-based methods, and 158 (16.6%) had a mass >100 kDa. The identified proteins were distributed across a wide pI range (4.31 to 11.78), and 8.6% of these proteins had pI >10 and 8.9% of these proteins had pI <5. Of the total identified proteins, 205 (21.5%) were unambiguously confirmed as PM proteins, 75 (36.6%) of which were predicted to have one or more predicted TMDs. We also analyzed the identified PM proteins on the basis of their calculated GRAVY value and predicted transmembrane domains. The GRAVY values of identified DRG PM proteins ranged from -1.131 to 0.954. Twenty-nine (14.2%) of 205 identified PM proteins had the positive values and can be considered to be hydrophobic (Fig. 4). Seventy-five (36.6%) identified PM proteins were integral membrane proteins with one or more predicted TMDs. These data indicate that, with our combined analytical strategies, the proteins with various physicochemical characteristics can be efficiently identified.

**Figure 5:**
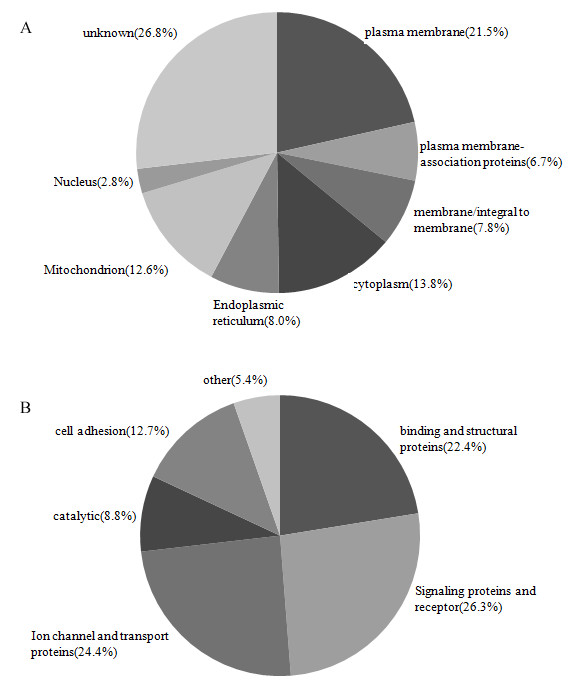
**Classification of the total proteins identified in rat DRG PM preparation**. (a) Classification of the identified proteins based on subcellular localization information. (b) Functional classification of the identified PM proteins.

In addition to the above-mentioned 205 PM proteins, 74 (7.8%) of the 954 identified proteins were categorized as membrane/integral to membrane proteins. Other identified proteins with a subcellular location annotation were annotated as PM-associated (6.7%), mitochondrial (12.6%), cytoplasmic (13.8%), endoplasm reticulum/golgi (8.0%), and nuclear (2.8%). Besides, 256 proteins (26.8%) had not a subcellular location annotation and were categorized as unknown proteins (Fig. 5A). It is worthy of mentioning that, in fact, the number of PM proteins identified in the present study should be greater than 205, because some of proteins annotated in general terms as 'membrane/integral to membrane proteins' were also PM proteins.

We also classified the 205 identified PM proteins according to their gene ontology (GO) function description. 26.3% were annotated as signal proteins and receptors, 24.4% as ion channel and transporters, 22.4% as binding and structural proteins, 12.7% as cell adhesion proteins, 8.8% as catalytic proteins. Other annotated proteins (5.4%) had biological activities such as protein folding and trafficking (Fig 5B). It could be seen that signal proteins, receptors, ion channel and transporters constituted more than half of the total identified PM proteins.

## Discussion

As a primary afferent neuron, DRG has complex signaling transduction systems. Of the identified PM proteins, large numbers of guanine nucleotide-binding proteins isoforms and Ras subfamily of GTPase were identified: Gα11, G(o)α, Gα12, G(q)α, G(t) α, Gβ2, G(g), G(i) α1, G(i) α2, Gβ4, G(s)α, G(I)/G(S)/G(T)β1, G(I)/G(S)/G(T)β2, Rab14, Rab18, Rab4b, Rab13, Rab11b, Rab5, Rab35, Rab10. These proteins have been implicated in a variety of processes, including signal transduction, neurotransmitter release, and membrane trafficking [16]. Due to the vesicle formation, traffic and membrane fusion, a number of Rabs can be found in PM other than intracellular membranes. For example, while initial studies showed that Rab5a regulates endocytic vesicle tethering and fusion, more recent evidence indicated that it also controls vesicle formation at PM and microtubule-dependent motility of endocytic structures [17,18]. Furthermore, several members of annexin family were detected, which play multiple roles in DRG cells. Annexin A1 and A3 were differently expressed in DRG sensory neurons [19]. Komori et al [5] observed that spinal nerve ligation up-regulated their expression. Annexin A1 as an acute-phase protein was indispensable for anti-inflammatory responses [20]. Flotillin-1 and flotillin-2 were also identified in the present study. Flotillin was used as a lipid raft marker and was demonstrated as a constitutive element in different signaling cascades [21]. Flotillin-1 was often used to evaluate the purity of PM fractions. Several receptors were identified, such as Taste receptor type 1 member 3 precursor, Cation-dependent mannose-6-phosphate receptor precursor. They were also the components of signaling pathways (Table 1, Additional file 1).

**Table 1:** Selected plasma membrane proteins identified from rat dorsal root ganglia

ID^a^	Proteins name	Scores/peptides	MW^b^	pI^c^	TMD^d^	GRAVY^e^
Ion channels						
IPI00211012	Voltage-gated potassium channel subunit beta-2	38/1	41280	9.09	0	-0.2692
IPI00198327	Voltage-dependent anion-selective channel protein 2	223/5	32353	7.17	0	-0.2210
IPI00190644	Potassium voltage-gated channel subfamily a member 1	96/3	56800	4.98	4	-0.0668
IPI00480697	Isoform 2 of transient receptor potential cation channel subfamily vmember	39/2	77382	6.27	6	0.0462
IPI00208249	chloride intracellular channel protein 4	172/5	28843	5.72	0	-0.4003
IPI00421874	Voltage-dependent anion-selective channel protein 1	580/11	32513	8.60	0	-0.4229
IPI00326646	Sodium channel	98/4	197410	6.30	16	0.1930
IPI00556929	Isoform 1 of voltage-dependent anion-selective channel protein 3	183/4	31292	8.90	0	-0.2964
IPI00327202	Aquaporin-1	77/2	29066	7.44	6	0.4881
IPI00767085	Similar to potassium channel tetramerisation domain containing protein12.	60/2	47077	9.35	0	-0.5641
Transporters						
IPI00192160	Plasmolipin	218/4	19934	9.45	4	0.9538
IPI00188119	Isoform long of potassium-transporting ATPase alpha chain 2	264/5	115656	6.07	8	0.0337
IPI00194875	Isoform wb of plasma membrane calcium-transporting ATPase 2	76/2	137922	5.65	8	-0.1662
IPI00205693	Sodium/potassium-transporting ATPase alpha-2 chain precursor	1529/32	113480	5.33	8	-0.0070
IPI00208061	Sodium/potassium-transporting ATPase subunit beta-3	199/4	32151	8.03	1	-0.2605
IPI00213585	Isoform short of calcium-transporting ATPase type 2c member 1	100/3	101519	6.18	8	0.1639
IPI00231267	Isoform a of plasma membrane calcium-transporting ATPase 1	131/3	139601	5.79	7	-0.1068
IPI00231451	Sodium/potassium-transporting ATPase alpha-3 chain	1389/28	113480	5.21	8	-0.0066
IPI00231462	Isoform short of potassium-transporting ATPase alpha chain 2	169/6	115656	5.79	8	0.1807
IPI00325847	Gpi-anchored ceruloplasmin	41/2	124469	5.35	0	-0.4821
IPI00326305	Sodium/potassium-transporting ATPase alpha-1 chain precursor	1913/37	114316	5.25	10	0.0020
IPI00339124	Sodium/potassium-transporting ATPase subunit beta-1	258/6	35762	8.81	1	-0.5241
IPI00390795	ATPase, Na^+^/K^+^transporting, alpha 4 polypeptide	398/8	115260	5.49	10	0.0337
IPI00476086	Atpase, h+ transporting, v0 subunit d isoform 1	290/9	40731	4.83	0	-0.0925
IPI00558343	Na^+^, K^+^-ATPase alpha-1 subunit (fragment)	93/2	27339	6.76	0	-0.1592
Receptors						
IPI00202168	Isoform 2 of sigma 1-type opioid receptor	100/3	21652	5.38	1	0.2942
IPI00203747	Low-density lipoprotein receptor precursor	95/3	100037	4.76	1	-0.4006
IPI00363550	Similar to transferrin receptor	112/5	86221	5.73	1	-0.2528
IPI00365669	Cation-dependent mannose-6-phosphate receptor precursor	52/2	31589	5.15	1	-0.1604
IPI00654429	Taste receptor type 2 member 110.	37/1	38552	10.0	0	0.8825
Cell-cell communication proteins						
IPI00476991	Neural cell adhesion molecule 1, 140 kda isoform precursor	273/8	95411	4.80	1	-0.4328
IPI00206054	Contactin-1 precursor	156/4	114278	5.71	0	-0.3094
IPI00208699	Contactin-associated protein 1 precursor	68/3	157765	6.49	1	-0.3064

^a^IPI database accession number. ^b^Calculated molecular weight. ^c^Value predicted by TMHMM. ^d^Gravy Value calculated using ProtParam algorithm. ^e^Calculated isoelectric point

Ion channels and transporters can mediate the movement of small molecules (ions and water) across the membrane bilayer, and some of them may be involved in the production of action potentials. In this study, the ion channels that we detected included Potassium voltage-gated channel subfamily a membrane 1, Voltage-gated potassium channel subunit beta-2, chloride intracellular channel protein 4 (CLIC4), Sodium channel, Voltage-dependent anion-selective channels protein 1 and water channel (Aquaporin-1), and so on. Of these, CLIC4 has recently attracted much attention. It was found that CLIC4 may interact with β-actin, dynamin, α-tubulin, and creatine kinase in the neuronal plasma membrane for cell motility inhibition and/or release of neuroactive molecules [22-24]. Additionally, Komori et al reported that spinal nerve ligation up-regulated the expression of CLIC4 [5]. At the same time, many important ion transporters were identified, such as members of plasma membrane calcium-transporting ATPase, potassium-transporting ATPase, plasma membrane calcium-transporting ATPase, Sodium/potassium-transporting ATPase, etc. (Table 1, Additional File 1). We also identified some transporters involved in nutrition metabolism, such as Solute carrier family 2, facilitated glucose transporter member 1. In addition, several other plasma membrane transport proteins, such as members of the AP-2 complex were identified. Clathrin adaptor AP2 and NSF interact with overlapping sites of GluR2 and play distinct roles in AMPA receptor trafficking and hippocampal LTD[25].

Of identified PM proteins from DRG cells, 26 (12.7%) were cell adhesion proteins, including members of integrin family, Contactin-1 precursor, Neural cell adhesion molecule 1, Isoform 2 of septin-9, etc. (Additional file 1). These proteins are known to participate in the formation of neuronal networks during development, specifically axon growth, synapse formation, and fasciculation [26]. Furthermore, some other important binding proteins such as Multidrug resistance protein 1 were also identified in the study. 6.7% of the identified proteins were cytoskeletal and extracellular proteins, which cross-link with PM. Some of them, such as neurofilament triplet L protein and neurofilament triplet M protein, are involved in the maintenance of neuronal caliber and stability.

The main contaminated proteins in PMEP were cytoplasmic and mitochondrial proteins, although their percentages in PMEP decreased compared to those in unwashed fraction. These contaminations may arise from subcellular organelles or cytoplasmic proteins closely contacted with the PM, or multiple localization within the cell [27]. Hsp47 classified as an ER protein was later discovered to be transported to the PM [28]. Voltage-dependent anion selective channel protein 1, also detected in the present study, was a neuron-specific protein expressed in PM and in mitochondria [29,30]. In addition, it is worth mentioning that, although the western blot analysis could not detected prohibitin, the marker enzyme of mitochondria, from PMEP under the experimental conditions, the final protein identification results indicated that the PMEP still contained some mitochondrial proteins. This result suggested that marker enzyme assay is not always reliable for the confirmation of subcellular organelle purity and other techniques should be additionally considered.

## Conclusion

The aqueous polymer two-phase partition is a simple, rapid, relatively inexpensive and highly reproducible method, without use of sophisticated equipment. Another advantage of the method was that small amount of tissue can be used for purification of PM. Using aqueous polymer two-phase partition in combination with high salt and high pH washing, a substantial enrichment of PM proteins were achieved. It is reasonable for the DRG PM to be enriched by using aqueous two-phase partition-based strategy as the preferred choice. In the present study, 205 PM proteins were unambiguously identified based on strict data validation and each of these proteins has its special role in the fundamental biological process of PM, including the exchange of materials and energy between cell and its environment, and signal transduction, etc. This work provided preliminary PM proteome data of rat DRG, which would help to our further understanding of the fundamental DRG functions.

## Materials and methods

### Materials

Trypsin (proteomics sequencing grade), dithiothreitol (DTT), iodoacetamide (IAA), 4-(2-hydroxyethyl)-1-piperazine ethanesulfonic acid (HEPES), protease inhibitor cocktail and sucrose were purchased from Sigma-Aldrich (St. Louis, MO). Acrylamide, bis-acrylamide, thiourea, urea, glycine, tris and sodium dodecyl sulfate (SDS) were from Amresco (Solon, OH, USA). Water was obtained with a Milli-Q Plus purification system (Millipore, Bedford, MA). All other reagents were domestic products of the highest grade available. Sprague-Dawley rats (weighting 125-200 g) were from Hunan Academy of Traditional Chinese Medicine (Changsha, China).

### Preparation of crude PM fraction

DRG were obtained from Sprague-Dawley rats according to the method described by Wang et al [31]. Briefly, twenty rats were anesthetized with ether and killed by decapitation. Their spinal columns were isolated and placed in Dulbecco's modified Eagle's medium (DMEM) saturated with oxygen. The spinal columns were cut out lengthways and the DRGs were separated from the spinal cords and other tissues. All steps were performed on ices. About 120 mg DRGs (wet weight) were homogenized in 4 ml of 250 mM sucrose, 15 mM Tris, pH 7.4, 0.1 mM phenylmethylsulfonyl fluoride and protease inhibitor cocktail with a Tissue Tearor (IKA products, T8 ultra-turrax, Germany) at 4°C. The homogenate was centrifuged at 600 × g for 10 min at 4°C to remove nuclei and unbroken cells. The pellet was homogenized and centrifuged again under the same conditions. The resulting supernatant was transferred to another tube and centrifuged at 100 000 × g for 1 h at 4°C (Beckman, Ti 70 rotor) to obtain a plasma membrane-contained fraction, which was named CPMF (Crude Plasma Membrane Fraction). After washing twice, CPMF was resuspended in 0.2 M potassium phosphate (pH 7.8) for further PM enrichment.

### Further enrichment of PM

The resuspended crude PM (CPM) was loaded onto a aqueous polymer two-phase system prepared from stock solution of 20% (w/w) Dextran T500 and 40% (w/w) polyethylene glycol 3350. All steps were performed at 4°C. The 16-g (6.4%) two-phase system was constituted of 5.12 g of 20% Dextran T500, 2.56 g of 40% poly (ethylene glycol) 3350, 0.4 ml of 0.2 M potassium phosphate (pH 7.2), 1.6 ml of 1 M sucrose. The weight of the system was brought to 14 g with distilled water [9]. Resuspended CPM was added to the two-phase system to a final weight of 16 g with distilled water and mixed 40 inversions. Phase separation was accelerated by a 5-min centrifugation at 1150 × g at 4°C. After phase separation, the system was divided into two phases: top phase and bottom phase. The top phase was removed to another tube, and the bottom phase was re-extracted with a fresh top phase (obtained from a two-phase system without adding CPMF). The obtained two top phases were combined and re-extracted twice with fresh bottom phase. To remove non-membrane proteins that adsorbed to membranes and further enrich PM proteins, the PMs obtained through two-phase partition were washed repeatedly with an ice-cold solution of 2 M NaCl, 10 mM HEPES/NaOH (pH 7.4), 1 mM EDTA (high salt washing), followed with 0.1 M Na_2_CO_3_, 1 mM EDTA, pH 11.3 (high pH washing) [32,33]. The obtained plasma membrane preparation was named PMEP, whose protein content was determined by using a Bio-Rad RC-DC protein assay kit.

### Western blot analysis

After separation by SDS-PAGE, the protein bands were electrotransferred to a PVDF membrane, which was blocked with 5% (v/v) non-fat dry milk in TBST (150 mM NaCl, 0.1% Tween-20, 25 mM Tris, pH 7.5) for 1 h at room temperature, followed by incubation with anti-Na^+^/K^+ ^ATPase antibody diluted 1:2000 or with anti-prohibitin antibody diluted 1:750 in the same TBST solution for 1 h at 4°C. After washing with TBST extensively, the membranes were incubated for 45 min at room temperature with the HRP-conjugated secondary antibody. After exposure to Hyperfilm ECL (Amersham), the enrichment of PM and the mitochondrion contamination were confirmed using the Quantity One 4.6.2 (Bio-Rad).

### SDS-PAGE and in-gel digestion

SDS-PAGE of the PMEP proteins was performed on a 4.8% stacking gel and a 11.5% separation gel. Prior to electrophoresis, PMEP containing 100 μg of proteins were resuspended in 0.2 ml sample loading buffer (0.5 M Tris-HCl, pH6.8, 4% SDS, 0.1 M DTT, 20% glycerol and a trace of bromophenol blue). After centrifugation at 12 000 × g for 12 min, the supernatant was loaded into gel wells. The SDS-PAGE was run at 20 mA on the polyacrylamide stacking gel and at 40 mA on the separating gel. After completion of electrophoresis, the separated protein bands were visualized using Coomassie brilliant blue G250. A low molecular weight calibration Kit (Bio-Rad) was used as the standard molecular weight marker.

For in-gel digestion, protein bands were excised from the SDS-PAGE gel and placed to clean Eppendorf tubes. In-gel digestion was performed as described previously [34]. After an overnight digestion, the gel pieces were extracted for 10 min in 100 μL 67% acetonitrile containing 0.1% formic acid with ultrasonication. The supernatants were pooled and lyophilized in a SpeedVac to about 5 μL for MS analysis.

### In-solution digestion

Proteins in PMEP were dissolved in 100 μL 8 M urea and 50 mM NH_4_HCO_3_, then reduced by 10 mM DTT at 50°C for 1 h and alkylated by 55 mM IAA at room temperature in the dark for1 h. After dilution to a urea concentration of 2 M with 25 mM NH_4_HCO_3_, the sample was digested with trypsin (1:50 w/w enzyme-to-protein ratio), and the mixture was incubated at 37°C overnight. After desalting on a C18 reverse-phase (RP) column (2.5 mm × 250 mm, 5 μm, Phenomenex) using a WATERS Alliance 2690 HPLC system, the sample was lyophilized to dryness for MS analysis.

### CapLC-MS/MS analysis

Tryptic peptides prepared as described above were analyzed by CapLC-ion trap mass spectrometry (Bruker Daltonics) coupled with an automated Agilent 1200 LC system equipped with an autosamlper and a C18 reverse-phase capillary column (PepMap,180 μm i.d., 15 cm long, LC-packings). Before separation on the reverse-phase capillary column, the sample was pre-concentrated on a C18 precolumn (500 μm i.d., 3.5 cm long, Bruker). When the sample was separated on the C18 PepMap column, the flow rate of eluting solution was 3 μL/min and the column temperature was set to 25°C. For the chromatography, the following solvents were used: solvent A (98% H_2_O, 1.9% acetonitrile, and 0.1% formic acid), solvent B (95% acetonitrile, 4.9% H_2_O, and 0.1% formic acid). The peptides eluted from the column were online directed into the mass spectrometry. The LC-MS system was controlled using Chemstation B01 (Agilent) and EsquireControlTM 6.1 (Bruker Daltonics) softwares. The nebulizer pressure was 10 psi. Drying gas flow rate was 5 L/min. Drying gas temperature was 300°C. Capillary voltage was 4000 V. The full MS scan mode was standard enhanced (m/z 350-1600 Da). Peptide ions were detected in MS scan, and seven most abundant in each MS scan were selected for collision-induced dissociation (MS/MS), using data-dependent MS/MS mode over the m/z range of 100-2000 Da.

### Data analysis and bioinformatics

Raw mass spectrometry data were processed, and Mascot compatible mgf files were created using Data AnalysisTM 3.3 software (Bruker Daltonics) with the following parameters: compounds (autoMS) threshold 10 000, number of compounds 300, retention time windows1.0 min. Searches were performed using Mascot software 2.0 (Matrixscience, London, U.K.), and the international protein index (IPI) rat database was used for protein identification. Search parameters were set as follows: enzyme, trypsin; allowance of up to one missed cleavage; peptide mass tolerance, 1.2 Da and MS/MS mass tolerance, 0.6 Da; fixed modification, carbamoylmethylation (C); variable modification, oxidation (at Met); auto hits allowed (only significant hits were reported); results format as peptide summary report. We confirmed candidate proteins according to the probability-based Mowse scores. Proteins were identified on the basis of distinct peptides whose ions scores exceeded the threshold, p < 0.05, which indicates identification at the 95% confidence level for these matched peptides. Most candidate proteins with mascot scores above the threshold were identified based on at least two identified peptides. For proteins identified by only one peptide with Mascot score exceeding the threshold, their MS/MS spectrum was systematically checked manually. For a protein to be confirmed, (i) the masses of all the major peaks (typically more than 7 peaks) in the MS/MS spectrum had to match those of the theoretically calculated fragment ions; (ii) the assignment had to be based on successive four or more b- or y-series ions; (iii) the molecular weight of the matched protein was in reasonable agreement with the gel migration data [35-37]. We kept to the principle of using the minimum set of protein sequences to account for all observed peptides. If several proteins were identified base on the same set peptides, only the one with more complete GO annotation was chosen.

The theoretical molecular weight and isoelectric point (pI) of identified proteins were retrieved from Mascot output files. The GRAVY values for identified proteins and peptides were analyzed using the ProtParam program http://tw.expasy.org/tools/protparam.html. Mapping of transmembrane domains (TMD) for the identified proteins was conducted using the TMHMM 2.0 program based on transmembrane hidden Markov model http://www.cbs.dtu.dk/services/TMHMM by submitting the FASTA files. The subcellular localization and function of identified proteins were retrieved by GO prediction and function annotations, respectively. Text-based annotation files were available for download from GO database ftp site at ftp://ftp.geneontology.org/pub/go.

## Abbreviations

DRG: dorsal root ganglion; PM: plasma membrane; CapLC: capillary column liquid chromatography; MS/MS: tandem mass spectrometry; CapLC-MS/MS: capillary column liquid chromatography coupled with tandem Mass spectrometry; SDS/PAGE: one-dimensional sodium dodecyl sulfate polyacrylamide gel electrophoresis; GO: gene ontology; PEG: polyethylene glycol; CPMF: crude plasma membrane fraction; PMEP: plasma membrane-enriched preparation; TMD: transmembrane domain; GRAVY: grand average of hydropathicity; DTT: dithiothreitol; IAA: iodoacetamide; HEPES: 4-(2-hydroxyethyl)-1-piperazine ethanesulfonic acid.

## Competing interests

The authors declare that they have no competing interests.

## Authors' contributions

XX carried out the proteomic studies, the data analysis and participated in design of the experiments; SH participated in experiments; HZ, JZL, JYS and LPJ participated in sample collection; JXX and LY participated in the identification of PM proteins; XCW and SPL were scientific lead and responsible for the experimental design, supervision and writing of the manuscript. All authors read and approved the final manuscript.

## Supplementary Material

Additional file 1 Information on identified proteins and peptides in rat DRG plasma membrane-enriched preparation. Click here for file
